# The relation between usage of an eHealth intervention for stress urinary incontinence and treatment outcomes: an observational study

**DOI:** 10.1186/s12875-024-02325-4

**Published:** 2024-03-16

**Authors:** Lotte Firet, Theodora Alberta Maria Teunissen, Rudolf Bertijn Kool, Reinier Peter Akkermans, Antoinette Leonarda Maria Lagro-Janssen, Huub van der Vaart, Willem Jan Jozef Assendelft

**Affiliations:** 1 Radboud University Medical Center, Department of Primary and Community Care, Research Institute for Medical Innovation, Postbox 9101, Geert Grooteplein 21, Nijmegen, 6500 HB the Netherlands; 2 Radboud University Medical Center, IQ Health Science Department, Nijmegen, the Netherlands; 3 Department of Gynecology, University Medical Center Utrecht, Utrecht, the Netherlands

**Keywords:** Urinary incontinence, eHealth, Pelvic floor muscle training, Primary care, Usage, Log data, Effectiveness

## Abstract

**Background:**

Stress urinary incontinence (SUI), though a prevalent condition among women, is undertreated in primary care. EHealth with pelvic floor muscle training is an evidence-based alternative to care-as-usual. It is unknown, however, how eHealth usage is related to treatment outcome, and this knowledge is required for general practitioners to implement eHealth in their practice. This study examines the relation between usage of eHealth for SUI and treatment outcomes by examining log data. Baseline factors were also explored for associations with treatment success.

**Method:**

In this pre-post study, women with SUI participated in “Baasoverjeblaas.nl”, a web-based intervention translated from the Swedish internet intervention "Tät®-treatment of stress urinary incontinence". Usage was based on log data and divided into three user groups (low, intermediate and high). Online questionnaires were sent before, after treatment and at six-months follow-up. The relation between usage and the primary outcome − treatment success (PGI-) − was studied with a binomial logistic regression analysis. Changes in the secondary outcomes − symptom severity (ICIQ-UI SF) and quality of life (ICIQ-LUTSqol) − were studied per user group with linear mixed model analysis.

**Results:**

Included were 515 users with a mean age of 50.5 years (12.0 SD). The majority were low users (*n* = 295, 57.3%). Treatment success (PGI-I) was reached by one in four women and was more likely in high and intermediate users than in low users (OR 13.2, 95% CI 6.1–28.5, *p* < 0.001 and OR 2.92, 95% CI 1.35–6.34, *p* = 0.007, respectively). Symptom severity decreased and quality of life improved significantly over time, especially among high users. The women’s expected ability to train their pelvic floor muscles and the frequency of pelvic floor muscle exercises at baseline were associated with treatment success.

**Conclusion:**

This study shows that usage of eHealth for SUI is related to all treatment outcomes. High users are more likely to have treatment success. Treatment success is more likely in women with higher expectations and pelvic floor muscle training at baseline. These findings indicate that general practitioners can select patients that would be more likely to benefit from eHealth treatment, and they can enhance treatment effect by stimulating eHealth usage.

**Trial registration:**

Landelijk Trial Register NL6570; https://onderzoekmetmensen.nl/nl/trial/25463.

**Supplementary Information:**

The online version contains supplementary material available at 10.1186/s12875-024-02325-4.

## Background

Pelvic floor muscle training (PFMT) via eHealth has been shown to be effective for women with stress urinary incontinence (SUI) and is an adequate alternative to care-as-usual [1, 2]. Conservative treatment options in primary care include instructions for PFMT by the general practitioner (GP), pelvic physical therapist or specialist nurse. Another treatment option includes wearing a pessary [3]. EHealth provides a solution for many affected women who presently go untreated in primary care [1, 4]. SUI is defined as the complaint of any involuntary urinary leakage on effort or on exertion or sneezing or coughing [5]. It affects one in four women and impairs quality of life by causing psychological problems and hampering physical mobility [6, 7]. However, help-seeking rates are low due to shame, unawareness of treatment options and SUI being considered as a normal part of ageing [8, 9]. Being effective self-management therapies that women can access at their own time and pace, eHealth applications with PFMT enhance access to care [10, 11]. GPs agree on this and acknowledge that such a self-management intervention is a solution for their own blind spot in detecting SUI [12, 13]. To actually prescribe eHealth, however, GPs report that they need to be convinced of the evidence for effectiveness of eHealth [13].

Although eHealth has the potential to supply treatment to a broader group of women with SUI, drop-out is a common issue, especially for eHealth with behavioral change interventions, which is indicated by the term “non-usage attrition” [14, 15]. Our previous report on the implementation of a web-based intervention for SUI showed that women used it with varying intensity, with the majority of them stopping prematurely [16]. Various reasons have been reported for termination of eHealth usage such as the absence of personal contact with a professional which could lead to motivational difficulties or to feelings of insecurity about the performance of PFMT[10, 12, 16, 17]. The question remains to what extent usage affects the effectiveness of an eHealth intervention. The most likely hypothesis is a dose–response curve, in which higher eHealth usage predicts a better treatment outcome. This is confirmed by eHealth studies of mental health problems [18], but other studies on eHealth usage found no relation with treatment outcome [15, 19]. Therefore, there might be other factors that influence the effectiveness of eHealth treatment. Early drop-out, for example, does not necessarily mean the absence of treatment effect [20]: our previous qualitative study showed that a subgroup of women with SUI reported they had stopped using eHealth after the first few modules because they had already gained a positive effect on their urinary leakage [16].

There is a lack of knowledge about the relation between usage and effectiveness of eHealth therapy for urinary incontinence. It is important to examine this relation because GPs need to know which patients are more likely to be successful and whether more intensive eHealth usage leads to better outcomes. This will facilitate GPs in selecting and supporting patients for eHealth therapy and stimulating their adherence. Currently, one observational study showed that higher self-reported usage of a mobile app correlated with improvement of symptoms [21].

Studying log data, however, would provide a more objective insight into the usage of an eHealth intervention [22]. This study, therefore, uses log data from participants in an eHealth intervention for SUI to answer the research question whether intensity of eHealth usage is related to three different treatment outcomes: treatment success (improvement); changes in symptom severity; and changes in quality of life. These outcomes were studied after the intervention period, which was three months, and at six months follow-up. Additionally, we explored if any baseline factors were associated with treatment success.

## Methods

### Design

A pre-post study design was performed to evaluate the effect of the eHealth intervention. All women took part in the intervention, and there was no comparison with a control group. The study protocol with an elaborate description of the methodology was published previously [23].

### Participants

Participants were recruited between July 2018 and March 2019 through traditional and social media. GPs in the university’s network were also asked to recruit women via leaflets or posters in waiting rooms. Women did not need a referral or an examination from a GP or another healthcare professional for participation, but they could register themselves on the website and provide digital informed consent. Eligibility was checked by the researcher through a brief questionnaire. Eligibility criteria are described in full in the published study protocol [23], but, in short, women were included if they were aged > 18 years with SUI or mixed urinary incontinence (MUI). MUI is the combination of SUI and urgency urinary incontinence, involuntary leakage accompanied by or immediately preceded by urgency [5]. The diagnosis was based on the Questionnaire for female Urinary Incontinence Diagnosis (QUID) [24]. A woman was considered to have SUI if she replied positively to the question: “Do you lose urine during quick moments such as coughing, sneezing, jumping or lifting something?”. A woman was considered to have MUI if she additionally replied positively to the question: “Do you feel such a strong need to urinate that you leak urine (even small drops) before reaching the toilet?”. Main exclusion criteria consisted of a diagnosis other than SUI or MUI, participation in another treatment program, or treatment (surgery or PFMT) or vaginal delivery in the last 6 months. Women who were included, received access to the website after completing the baseline questionnaire. There was no reimbursement for participation, nor did participants have to pay.

### Intervention

The eHealth intervention “*Baas over je blaas*” is a Dutch translation of the internet-program "Tät®-treatment of stress urinary incontinence" from the eContinence group of Umeå University, Sweden [25]. They showed its effectiveness in various RCTs [26, 27]. Our Dutch research group gained permission to use it for research purposes through a non-commercial license agreement.

**Fig. 1:**
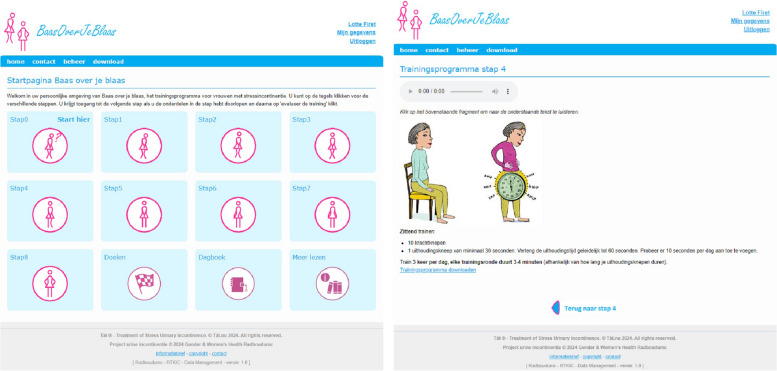
Screenshot of home page with eight modules (left) and training program 4 (right)

The eHealth intervention consisted of PFMT that was addressed in eight escalating modules (Fig. 1). The modules contained a maximum of three components: background information, the training program with pelvic floor muscle exercises and a test exercise. All information was provided by text, illustrations and audio fragments and could be downloaded as a PDF file. The test exercise served as a check for women to know whether they could continue to the next module. To gain access to the next module, women had to fill in a training report at the end of each module. Depending on the module number, women were advised to train two to three times a day for at least one week, but they could do the training at their own time and pace. Access to the intervention was closed after three months. This is in line with the Dutch primary care guideline, which suggests assessing the effectiveness of PFMT after three months [3]. In addition, an intervention period of three months enables comparison with the Swedish intervention "Tät®-treatment of stress urinary incontinence" [26]. During the whole intervention, there was no organized contact with a healthcare professional, but e-mail contact with a researcher (LF; GP trainee and PhD student) was available for both content-related and technology-related questions. To stimulate adherence to the training, a maximum of two e-mail reminders was sent if a woman had not logged in for one week. As part of the information provided in the intervention, women were advised to consult their GP if they made no progress or if they were unable to perform the exercises.

### Data collection

#### Outcome measures

The primary treatment outcome in this study is treatment success. Treatment success is defined as “much” or “very much” improved on the Patient Global Impression of Improvement (PGI-I) question [28]. Secondary outcomes are symptom severity (ICIQ-UI SF) and quality of life (ICIQ-LUTSqol and SF-12) [29–31].

#### Questionnaires

All participants completed an online questionnaire at baseline (T0), and regardless of their progression in the eHealth intervention, all women were sent follow-up questionnaires. The first questionnaire (T1) was sent after access to the intervention had stopped, which was three months after baseline, and the second questionnaire (T2) was sent six months after baseline. Women who filled in the T1 and T2 questionnaires were named “completers”. The questionnaires were independent from each other, so it was possible for a woman to complete T2 if she had not completed T1. An extensive description of the questionnaires was published previously [23].

The questionnaire at T0 collected demographical data (age, education), medical items (parity, postmenopausal status) and incontinence-related items (incontinence type (SUI or MUI), duration (shorter or longer than five years), previous PFMT via therapist (yes/no), previous contact for urinary incontinence with healthcare professional (yes/no), frequency of pelvic floor muscle exercises at baseline (never, < 1/week, > 1/week) and expectations about treatment results and about the ability to train pelvic floor muscles). Symptom severity (ICIQ-UI SF) and quality of life (ICIQ-LUTSqol and SF-12) were assessed in all questionnaires. Treatment success (PGI-I) was collected at T1 and T2.

Educational level was dichotomized into low (primary and lower secondary education) versus high (upper secondary up to doctoral equivalent level). Expected treatment results were divided into three categories (slight improvement, major improvement or cure), and this variable was adapted from another study [32]. The expected ability to train the pelvic floor muscles was assessed on a 10-point scale ranging from very low to very high expectations [33].

Treatment success was assessed by the PGI-I, which is a validated question asking participants to rate their current urinary incontinence compared to prior to treatment [28]. It has seven response options, ranging from “very much better” to “very much worse”, with “much better” or “very much better” being defined as success.

Symptom severity was assessed by the validated International Consultation on Incontinence Questionnaire Short Form (ICIQ-UI SF) [29]. This is a 6-item questionnaire, assessing frequency, amount of leakage and impact of urinary incontinence on daily life [29]. Scores range from 0 to 21, with a higher score corresponding with a higher symptom severity. Based on these scores, women can be divided into four categories (1–5 = slight, 6–12 = moderate, 13–18 = severe, 19–21 = very severe).

Quality of life was assessed by the condition-specific Lower Urinary Tract Symptoms Quality of Life (ICIQ-LUTSqol) questionnaire [30], which contains 19 items about condition-specific issues, such as physical and social limitations relating to incontinence. Scores range from 19 to 76, with a higher score implying a greater impact on quality of life [30]. The Short-Form (SF-12) assesses general quality of life and comprises a subset of 12 questions from the SF-36 assessing physical and mental well-being with the Physical Component Summary (PCS-12) and Mental Component Summary (MCS-12). Scores range from 0 to 100, with a higher score corresponding to better physical and mental health [31].

#### Log data

**Fig. 2:**
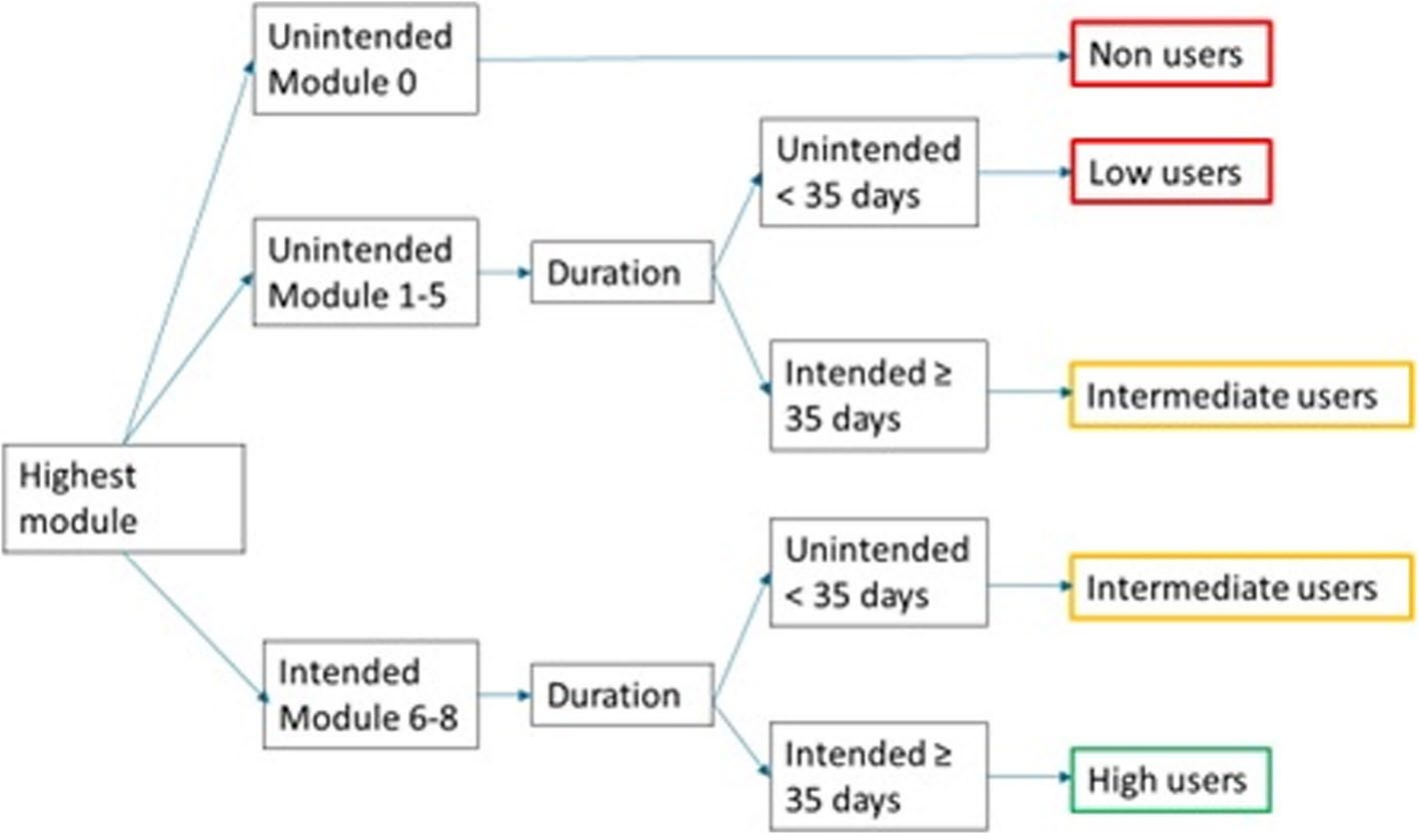
Flowchart of user groups based on module number and duration

Usage was assessed by the log data that were collected during the three months when women had access to the eHealth intervention. The full description and results of three usage parameters (module number, frequency and duration) have been reported previously [16]. Usage was defined by three user groups (low, intermediate and high users), which were created on the basis of the usage parameters of module number and duration (Fig. 2). The module number was the module that a participant had reached when access to the website was terminated. Duration was the total number of days between the first login and the date on which the last training report was completed. For women who dropped out in module number one and who, therefore, did not complete a training report, duration was defined as the difference between the first and the last login.

For both parameters, the term “intended use” was applied, which were predefined cut-off points that a participant should reach to benefit from the intervention. “Intended use” for module number was at least module five, when all exercises would have been addressed. “Intended use” for duration was at least 35 days, which is the multiplication of five modules with the recommend training duration of at least one week.

### Data analysis

Descriptive statistics were calculated for baseline variables and for the treatment outcomes at T1 and T2. For continuous variables, mean and standard deviation (SD) or median and interquartile range were determined. For categorial variables, number and percentages were determined. Participants who completed a questionnaire after the date of expiry (> 120 days after baseline for T1; > 210 days after baseline for T2) were excluded from analyses for the measurement concerned. Differences in baseline variables between completers and non-completers of the questionnaires and between user groups were tested with an independent *t* test or Mann–Whitney U test for continuous variables and with a Chi-square test for categorical variables. Binomial regression analysis was used to study the relation between usage and treatment success (PGI-I).

Two models were constructed for both T1 and T2: (1) an unadjusted model with usage in a univariate regression analyses and (2) an adjusted model with corrections for factors that were potential confounders. Based on literature and on our previous findings, these factors included age, symptom severity (ICIQ-UI SF), previous PFMT, expected treatment results and expected ability to train pelvic floor muscles [16, 32, 33]. We checked for effect modification by applying interaction terms in the model and we reported effect modification if the regression coefficient at issue was significant (*P* < 0.05). Regression coefficients of unadjusted and adjusted models were compared, and in the case of more than ten percent deviation, we would conclude that confounders had affected the relationship between usage and treatment success.

Linear mixed regression models were used to analyze changes in symptom severity (ICIQ-UI SF) and quality of life (ICIQ LUTS-QoL and SF-12) across time for different user groups. We checked if there was selective drop-out on these outcomes at T1 and T2. Two models were compared: (1) unadjusted models and (2) models adjusted for baseline differences between completers and non-completers and for baseline differences between user groups. No imputations were used for missing data because mixed models deal with them adequately [34].

Univariate logistic regression analysis was performed to analyze if there were baseline characteristics, other than usage, that were associated with treatment success at T1 and T2. These characteristics included age, incontinence type, duration, severity (ICIQ-UI SF), previous PFMT, frequency of pelvic floor muscle exercises at baseline, expected treatment results and expected ability to train pelvic floor muscles. Variables with a significance level of *P* < 0.2 were included in the multivariate model. Variables were excluded stepwise in order of the highest *P* value until only statistically significant (*P* < 0.05) variables remained. A value of *p* < 0.05 was considered statistically significant for all analyses, based on two-sided testing. Data were analyzed using IBM SPSS version 25 (SPSS, IBM Corp., Armonk, NY).

## Results

### Baseline characteristics

**Fig. 3:**
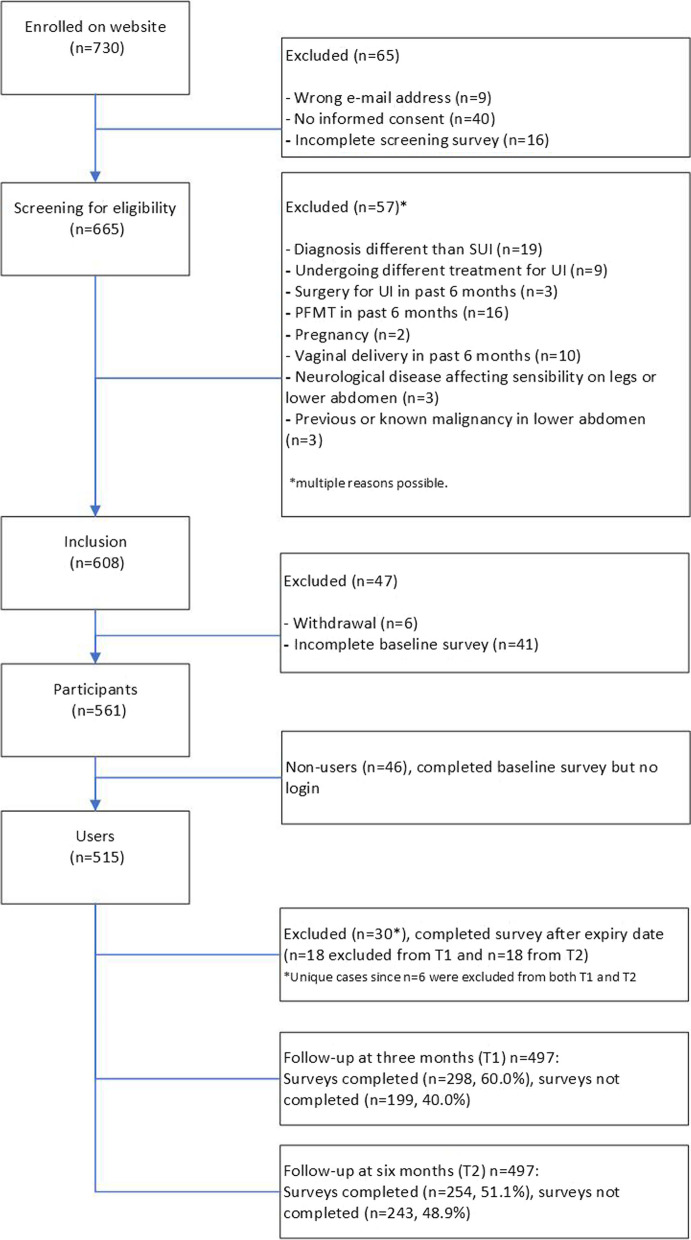
Flowchart of the study

Out of a total of 730 women who enrolled on the website, 515 users (70.5%) started with the intervention (Fig. 3). Women were excluded mainly because they never logged in (*n* = 46) or did not complete the baseline survey (*n* = 41). T1 and T2 questionnaires were completed by 298 (57.9%) and 254 (49.3%) users, respectively. The mean age was 50.5 years (12.0 SD, range 21–81 years), and the majority were highly educated (*n* = 469, 91.1%) (Table 1). Most women had a moderate severity of urinary incontinence (*n* = 357, 69.3%). The majority (*n* = 362, 72%) did not receive previous PFMT from a professional. Completers of T1 and T2 questionnaires differed from non-completers in various characteristics (Table S1-S2): completers were older, more often postmenopausal, more frequently performed pelvic floor muscle exercises at baseline and were more often intermediate or high users of the eHealth intervention compared to non-completers. There was no selective drop-out on the outcome variables (ICIQ-UI SF, ICIQ LUTS-QoL or SF-12).

**Table 1:** Baseline characteristics for all users

**Outcome variable**	*n* = 515 (100%)
**Demographic**	
Age, years, mean (SD)	50.5 (12.0)
Educational level, n (%)	
• Low	46 (8.9)
• High	469 (91.1)
**Medical history**	
Parity > 1 child(ren), n (%)	436 (84.7)
Postmenopausal, n (%)	
• Yes	242 (47.0)
• No	230 (44.7)
• Unknown	43 (8.3)
General quality of life (SF-12), mean (SD)	
• Physical health (PCS-12)	50.8 (7.4)
• Mental health (MCS-12)	49.2 (9.0)
**Incontinence related**	
Incontinence type, n (%)	
• SUI	425 (82.5)
• MUI	90 (17.5)
Duration < 5 years, n (%)	293 (56.9)
Severity of incontinence (ICIQ-UI SF), mean (SD)	9.9 (3.2)
Severity of incontinence (ICIQ-UI SF), n (%)	
• Slight	39 (7.6)
• Moderate	357 (69.3)
• Severe	119 (23.1)
• Very severe	0 (0)
Quality of life (ICIQ LUTS-QoL), mean (SD)	32.0 (6.9)
Previous PFMT via therapist^a^, n (%)	145 (28.2)
Previous contact with healthcare professional^a^, n (%)	195 (37.9)
Frequency of pelvic floor muscle exercises at baseline, n (%)	
• Never	231 (44.9)
• < 1/week	218 (42.3)
• > 1/week	66 (12.8)
Expected ability to train pelvic floor muscles, mean (SD)	7.7 (1.4)
Expected treatment results^a^, n (%)	
• Slight improvement	85 (16.5)
• Major improvement	374 (72.8)
• Cure	55 (10.7)

^a^Missing values for the variables previous PFMT via therapist, previous contact with healthcare professional and expected treatment results (*n* = 4, *n* = 8, *n* = 1, respectively)

### Characteristics of user groups

Users (*n* = 515) were divided into three user groups, the majority of whom were low users (*n* = 295, 57.3%), followed by intermediate users (*n* = 133, 25.8%) and high users (*n* = 87, 16.9%). When user groups were compared for characteristics at baseline, they differed in age, postmenopausal status, previous PFMT for incontinence, frequency of pelvic floor muscle exercises at baseline and expected ability to train pelvic floor muscles (Table 2). Compared to low and intermediate users, high users were older, more often postmenopausal, had more often performed previous PFMT, had a higher frequency of pelvic floor muscle exercises at baseline and had higher expected ability to train pelvic floor muscles. These differences were comparable for T1 and T2.

**Table 2:** Baseline characteristics per user group

Outcome variable	Low Users n (%) 295 (57.3)	Intermediate users n (%) 133 (25.8)	High users n (%) 87 (16.9)	Comparison (*P* value)
**Demographic**				
Age, years, mean (SD)	48.3 (12.0)	52.1 (11.6)	55.3 (10.9)	0.001*
Educational level, n (%)				0.95
• Low	27 (9.2)	11 (8.3)	8 (9.2)	
• High	268 (90.8)	122 (91.7)	79 (90.8)	
**Medical history**				
Parity 1 > child(ren), n (%)	245 (83.1)	117 (88.0)	74 (85.1)	0.42
Postmenopausal, n (%)				0.001*
• Yes	120 (40.7)	67 (50.4)	55 (63.2)	
• No	145 (49.2)	54 (40.6)	31 (35.6)	
• Unknown	30 (10.2)	12 (9.0)	1 (1.1)	
General quality of life (SF-12), mean (SD)				
• Physical health (PCS-12)	50.3 (7.6)	50.6 (7.7)	52.5 (5.8)	0.05
• Mental health (MCS-12)	48.6 (9.4)	50.4 (8.0)	49.5 (8.6)	0.16
**Incontinence related**				
Incontinence type, n (%)				0.55
• SUI	245 (83.1)	106 (79.7)	74 (85.1)	
• MUI	50 (16.9)	27 (20.3)	13 (14.9)	
Duration < 5 years, n (%)	164 (55.6)	78 (58.6)	51 (58.6)	0.79
Severity (ICIQ-UI SF), mean (SD)	10.2 (3.2)	9.5 (3.2)	9.6 (2.9)	0.09
Quality of life (ICIQ LUTS-QoL), mean (SD)	32.3 (7.1)	31.5 (7.0)	31.4 (5.8)	0.38
Previous PFMT via therapist^a^, n (%)	85 (29.1)	28 (21.2)	32 (36.8)	0.04*
Previous contact with healthcare professional^a^, n (%)	116 (40.1)	44 (33.3)	35 (40.7)	0.37
Frequency of pelvic floor muscle exercises at baseline, n (%)				< 0.001*
• Never	157 (53.2)	49 (36.8)	25 (28.7)	
• < 1/week	112 (38.0)	63 (47.4)	43 (49.4)	
• > 1/week	26 (8.8)	21 (15.8)	19 (21.8)	
Expected ability to train pelvic floor muscles, mean (SD)	7.5 (1.4)	7.7 (1.4)	8.1 (1.5)	0.006*
Expected treatment results^a^, n (%)				0.09
• Slight improvement	52 (17.7)	24 (18.0)	9 (10.3)	
• Major improvement	218 (74.1)	93 (69.9)	63 (72.4)	
• Cure	24 (8.2)	16 (12.0)	15 (17.2)	

^a^Missing values for the variables previous PFMT via therapist, previous contact with healthcare professional and expected treatment results (*n* = 4, *n* = 8, *n* = 1, respectively). *Significance level *P* <0.05

### Usage and treatment success

Around one in four women reported treatment success (PGI-I), 26.2% at T1 and 25.2% at T2, the majority of these being high users (Table 3). Most women had no treatment success according to the applied definition but reported they had little improvement after eHealth participation (39.9% at T1 and 38.6% at T2). Usage was related to treatment success at T1 and T2 (*P* < 0.001) after performing a logistic regression model. High and intermediate users were more likely to have treatment success than low users (Table 3). After correction for confounders (age, symptom severity, previous PFMT, expected treatment results and expected ability to train pelvic floor muscles), the regression coefficients in the unadjusted model did not change. Use of interaction terms revealed no effect modification.

**Table 3:** Unadjusted odds ratios for the relation between usage and treatment success at T1 and T2

	Treatment success T1 n (%)	Unadjusted OR T1 (95% CI)	*P* value	Treatment success T2 n (%)	Unadjusted OR T2 (95% CI)	*P* value
**User group**			< 0.001			< 0.001
• Low	11 (14.1)	Ref		10 (15.6)	Ref	
• Intermediate	23 (29.5)	2.9 (1.4–6.3)	0.007	24 (37.5)	3.3 (1.5–7.3)	0.004
• High	44 (56.4)	13.2 (6.1–28.5)	< 0.001	30 (46.9)	5.7 (2.6–12.8)	< 0.001
**Total group**	78 (26.2)			64 (25.2)		

### Usage and symptom severity

**Fig. 4:**
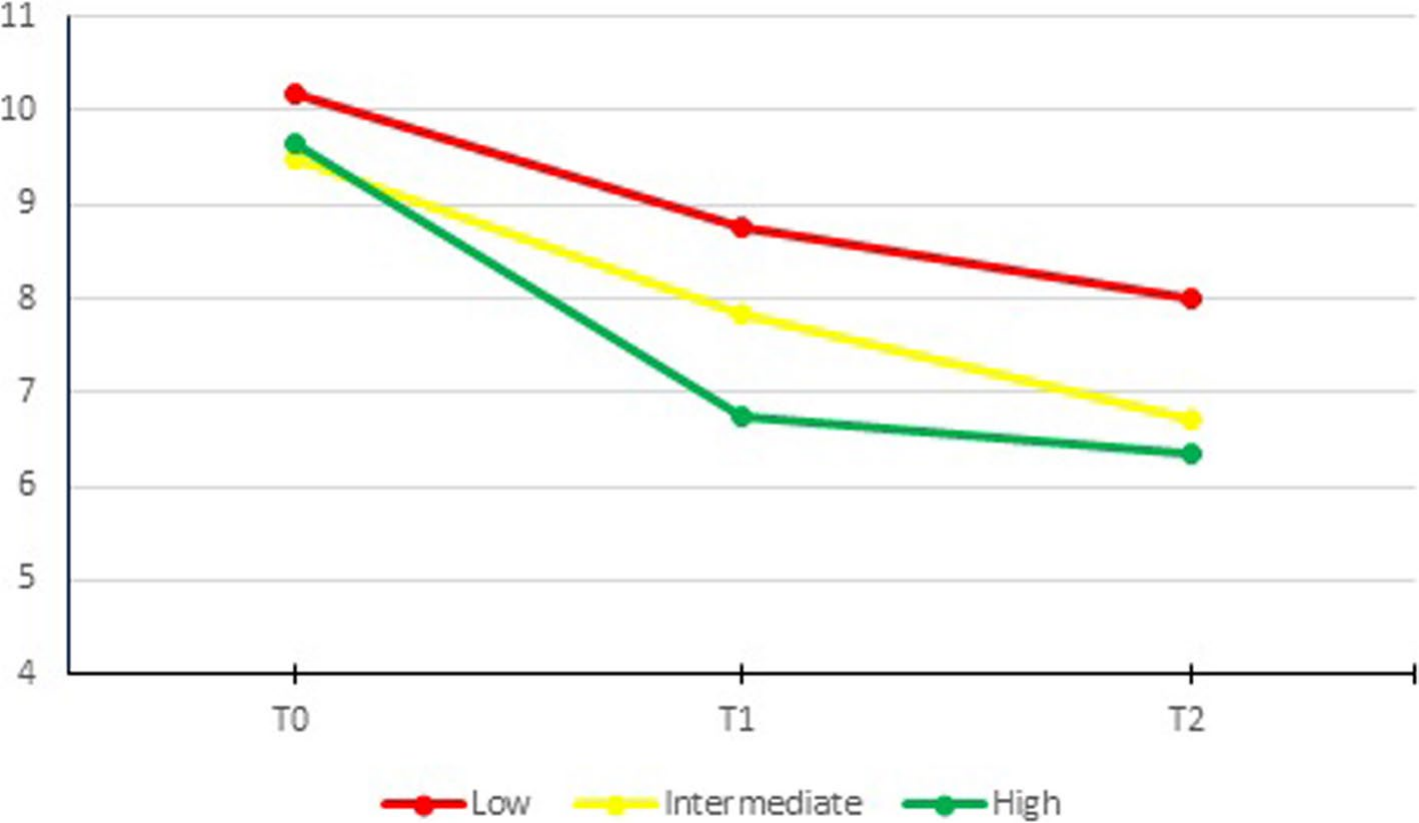
Symptom severity (ICIQ-UI SF) over time per user group

There was an overall significant difference in change over time for symptom severity between the user groups, *P* = 0.001. At baseline, scores per user group were 10.2 (95% CI 9.8, 10.5), 9.5 (95% CI 9.0, 10.00) and 9.6 (95% CI 9.0, 10.3) for low, intermediate and high users, respectively. There was a significant difference between low and intermediate users (*P* = 0.044) at baseline (Fig. 4). After the intervention (T1), high users had a significantly greater decrease in scores than low users (-1.5 [95% CI -2.2, -0.8], *P* < 0.001). ICIQ-UI SF scores were 8.8 (95% CI 8.3, 9.3) and 6.8 (95% 6.1, 7.5), for low and high users, respectively.

At follow-up (T2) this difference in scores remained significant, with a greater decrease in scores for high than for low users -1.1 (95% CI -1.9, -0.3), *P* = 0.005. ICIQ-UI SF scores were 8.0 (95% CI 7.5, 8.5) and 6.3 (95% 5.6, 7.0), for low and high users, respectively. When we compared intermediate and high users, high users had a significantly greater decrease in scores than intermediate users -1.2 (95% CI -2.0, -0.5), *P* = 0.002, but only at T1.

There were no significant differences between low and intermediate users. These effects remained the same when the models were corrected for variables that differed between completers and non-completers of the questionnaires (age, postmenopausal status, previous PFMT for incontinence, PFMT frequency at baseline and the expected ability to train pelvic floor muscles) (Table S3a).

### Usage and quality of life

**Fig. 5:**
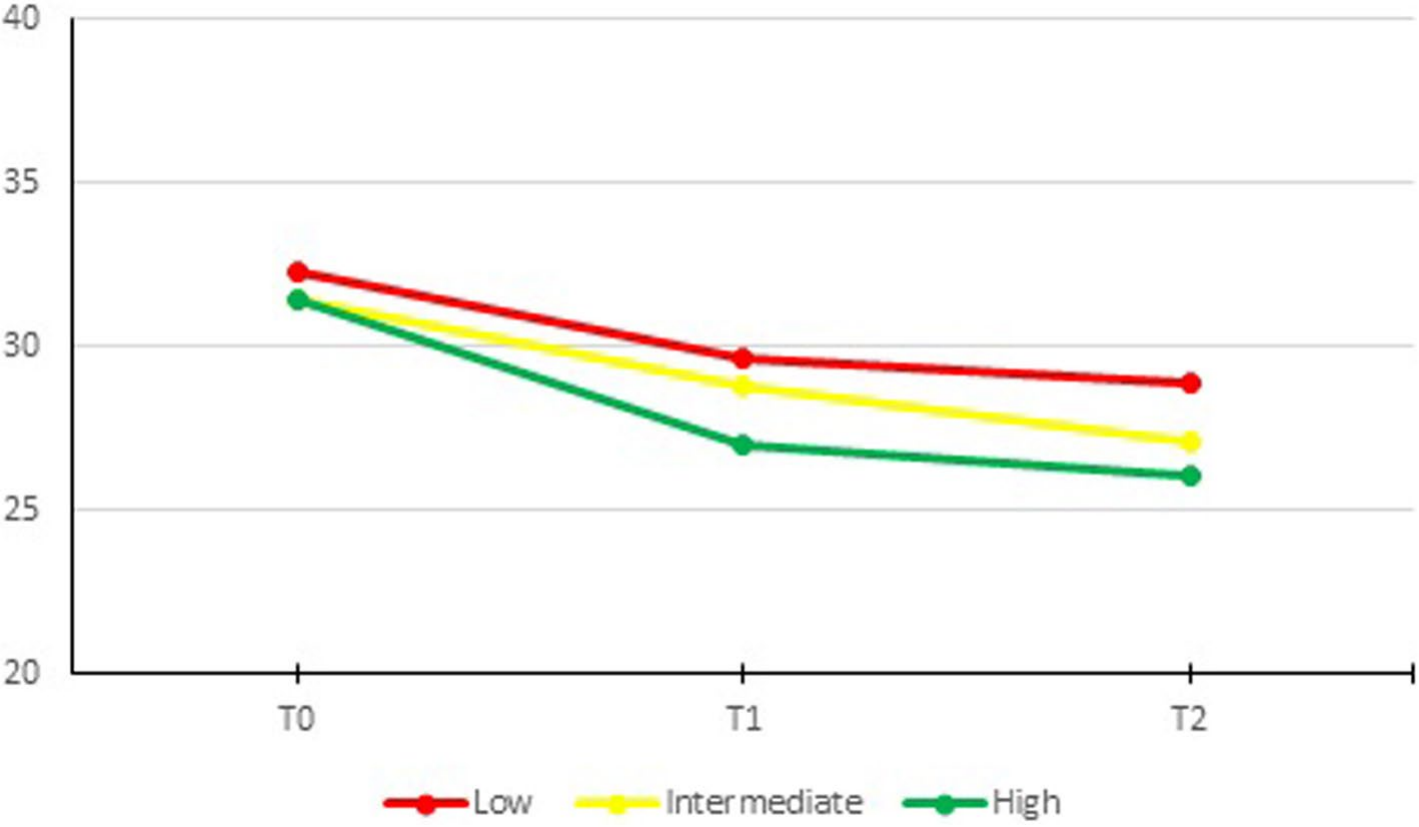
Quality of life (ICIQ LUTS-QoL) over time per user group

There was an overall significant difference in change of condition-specific quality of life over time between user groups, *P* = 0.012. At baseline scores were 32.3 (95% CI 31.6, 33.1) vs 31.5 (95% CI 30.3, 32.6) vs 31.4 (95% CI 30.0, 32.8) for low, intermediate and high users, respectively (Fig. 5). After the intervention (T1), high users had a significantly greater decrease in scores than low users (-1.7 (95% CI -3.0, -0.4), *p* = 0.009). Mean scores for low and high users were 29.6 (95% CI 28.7, 30.6) and 27.0 (95% CI 25.6, 28.5) at T1.

At follow-up (T2), high users had a significantly greater decrease in scores than low users (-2.0 (95% CI -3.3, -0.6), *p* = 0.004). Mean scores for low and high users were 28.9 (95% CI 27.9, 30.0) and 26.0 (95% CI 24.6, 27.5) at T2. When comparing intermediate and high users, there was a significant difference in decrease at T1 (-1.7 (95% CI -3.1, -0.4), *P* = 0.011), but not at T2.

There were no significant differences between low and intermediate users. These effects remained the same when the models were corrected for variables that differed between completers and non-completers of the questionnaires (age, postmenopausal status, previous PFMT for incontinence, PFMT frequency at baseline and the expected ability to train pelvic floor muscles) (Table S3b). The general quality of life on the SF-12-PCS and SF-12-MCS scales showed no significant decrease in time.

### Factors associated with treatment success

“Frequency of pelvic floor muscle exercises at baseline” and “expected ability to train the pelvic floor muscles” both significantly contributed to the multivariate model. An exercise frequency of once a week or more at baseline was more likely to result in successful treatment compared to not training at all (OR 3.3, 95% 1.5–7.1, *p* = 0.003). Having higher expectations of the ability to train the pelvic floor muscles was associated with treatment success (OR 1.6, 95% 1.3–2.0, *p* < 0.001). For the model at T2, only the expected ability to train the pelvic floor muscles appeared to be associated with treatment success (OR 1.5, 95% CI 1.2–1.9, *p* < 0.001). The frequency of pelvic floor muscles exercises at baseline was not selected for the model from univariate analyses (Table S4-S5). The variables in the models for T1 and T2 explained 14.2% and 7%, respectively, of the variability in treatment success (Nagelkerke R square).

## Discussion

### Principal findings

This study shows that longer or more intensive usage of an eHealth intervention for SUI leads to better treatment outcomes. High users are more likely to have treatment success, their symptom severity decreases to a greater extent and their quality of life improves significantly more compared to low users. In addition, treatment success is more likely among women with higher expectations about their ability to train their pelvic floor muscles and among those who already frequently performed pelvic floor muscles exercises at baseline.

### Comparison with literature

To the best of our knowledge, this is the first study that shows a dose–response relation between usage of an eHealth intervention for urinary incontinence and clinical effects. Positive associations between usage and treatment outcome were also found for digital interventions for mental health and physical health, such as weight management and smoking cessation [18, 35, 36]. However, this association was not found for eHealth for cardiovascular disease management and was inconsistent for dietary intake [37, 38]. It is difficult to derive underlying explanations from the studies on these various indications as these studies are heterogenous not only in targeted health conditions but also in how they operationalize and assess usage.

A systematic review showed that the relation with treatment outcome in eHealth interventions depended on the type of usage parameter that was studied (e.g., login frequency, time spent in the intervention, modules completed) [36]. We defined usage by the parameters “duration” and “modules completed” and found a positive association with treatment outcome. This is in line with other studies that used “module completed” as their usage parameter [18, 35, 36]. The fact that users need to actively complete an element of the eHealth intervention is possibly a requirement for reaching treatment effect.

This study shows that eHealth usage is an important aspect when analyzing treatment outcome. Usage fits into the overarching theme of “engagement”, which is a prerequisite for effecting change in health outcomes. Engagement is important especially for interventions that target behavior change, which, for SUI, means integrating PFMT into daily life. Researchers propose to understand engagement on a micro- and macro-level [15, 39], where micro level is the moment-to-moment user-interaction with the intervention, and macro level is how users identify with the goals targeted by the intervention. Depending on timing, context and individual factors, the importance of these levels varies [15].

When we apply this concept to eHealth for urinary incontinence, micro-level engagement (e.g., logging in on the website and completing modules) could be important initially with macro-level engagement (e.g. performing PFMT, adherence) gradually becoming more important. This study focused mainly on micro-level engagement but did not report how women adhered to PFMT during and after the intervention in their daily lives. Our previous report showed adherence levels of sixty percent for the remaining users in every other module, but there were no follow-up adherence data [16]. To understand the effect of eHealth on urinary incontinence, it would be of added value to combine these data − usage of eHealth and adherence to PFMT − as being reflective for micro- and macro-level engagement.

Regarding treatment outcomes, this study shows that one in four women reached treatment success and two-thirds reported any improvement, which is in line with a Dutch RCT on an app-based intervention for urinary incontinence [2]. However, a Swedish RCT with the same web-based intervention ("Tät®-treatment of stress urinary incontinence"), reported a higher success rate of forty percent [26]. Women in this study had contact with a urotherapist throughout the training, who stimulated them to practice and provided them with the login codes for every next module. This contact may have increased eHealth usage, thus increasing the success rate.

Other treatment outcomes in this study were symptom severity (ICIQ-UI SF) and quality of life (ICIQ-LUTSqol), and improvements on these scores were comparable with the Dutch RCT [2]. Improvements were greater for high users than for low users, and this was clinically relevant for the high users as it has previously been established that the minimum important difference is 2.5 for the ICIQ-UI SF and 3.7 for ICIQ-LUTSqol [40]. However, intermediate and low users also improved after participating in eHealth, which might imply that a placebo effect could have occurred or that patients were influenced by unknown factors. Trials on eHealth for urinary incontinence also showed an improvement in the control group, albeit to a lesser extent than in the intervention group [26, 41].

### Implications for practice

Our findings on the relation between eHealth usage and treatment outcome have important implications for further implementation in primary care and are directly applicable to clinicians, researchers, patients and other people involved in implementing eHealth for SUI. First, this study contributes to the evidence that eHealth is an effective treatment option for urinary incontinence by showing that users need to persist to gain greater effect. It is known that healthcare professionals still need to be persuaded by evidence of the effectiveness of eHealth for UI as a condition for them to implement it in their daily practice [12, 13].

Secondly, the study shows that it is highly relevant to invest in avoiding non-usage attrition. GPs, or other primary care professionals, can play a crucial role in increasing treatment effect by motivating women to continue with the eHealth intervention [10, 12]. As the absence of contact was a barrier to using eHealth, contact with a GP or practice nurse throughout the training program will meet women’s need for personal contact [16, 17]. Other methods to stimulate the use of eHealth might be through technological tools within the eHealth intervention itself [42], such as a chat function with a healthcare professional, tailored reminders or improved visual materials.

A third implication for practice is that GPs can better predict which patients are more likely to benefit from eHealth. In line with another study [33], women’s baseline expectations of their ability to perform the training are associated with treatment success: the higher the expectations, the more likely the success of the program. GPs could help women feel they are able to perform the training program by using techniques from motivational interviewing. They could, for example, check their patients’ trust by asking them: “On a 10-point scale, how convinced are you that you will be able to keep using eHealth and perform PFMT?”. Or they could ask their patients’ consent to get back to them on this topic in one or two weeks, a question that could support progress into the next phase of Prochaska and DiClemente’s stages of behavioral change [43]. Additionally, GPs could take baseline performance of pelvic floor muscle exercises into account as women who frequently perform exercises at baseline are more likely to have eHealth treatment success [10].

Age, finally, did not appear to be associated with treatment success. This implies that GPs can advise both younger and older women to use eHealth. Our previous study, however, showed that women aged 50 + are more likely to be high users [16], and other studies have shown that higher age is a predictor of treatment success [32, 33]. Although it remains unclear how age and treatment outcome are related, it is apparent that women of all ages can participate in eHealth for SUI.

### Strengths and limitations

A major strength of the study is that log data were collected to study the usage of eHealth. Log data provide an objective insight into how people interact with the technology and avoids reliance on self-report data [22]. For treatment outcomes, recommended and validated questionnaires were used, which enables comparison with other studies. This study had a large sample size, which strengthens our conclusions.

There are some limitations to this study. First, the absence of a control arm has several implications. It limits the conclusions about the treatment effects being caused by the intervention or by other factors. Nor could we rule out that our selection of participants influenced the positive treatment outcome. Another implication of the absence of a control group is that we could not rule out that regression to the mean has taken place for the secondary outcomes (symptom severity and quality of life). However, results about these treatment outcomes are in line with previous RCTs that had both an intervention and a control group [2, 26].

The response rate on the follow-up questionnaires was reasonable, nearly sixty and fifty percent for T1 and T2 respectively. However, completers of the questionnaires were older and they performed pelvic floor muscle exercises at baseline more frequently compared to non-completers. Therefore, we could not rule out that nonresponse bias occurred. Another limitation is the small number of low-educated participants (9%), which hampers the generalizability of the results as the proportion of low-educated people in the Dutch population is 29 percent [44]. However, this is not exceptional in this research field [2, 26, 41] and might be related to higher literacy levels among eHealth users [45], but also among participants of eHealth research.

Finally, a limitation of this study is that the treatment outcomes rely on self-reported data solely. It would strengthen the results if women were assessed by a trained specialist regarding urinary incontinence. In the Netherlands, this could be a GP or a specialized pelvic physiotherapist in primary care. These professionals could assess the strength of the pelvic floor muscles and check whether the right muscles are contracted.

## Conclusion

This study shows a positive relation between the level of usage of an eHealth intervention for SUI and treatment outcomes. Additionally, women’s expectations about their ability to perform the training and baseline frequency of pelvic floor muscle exercises are associated with treatment success. General practitioners can tell their patients that eHealth for urinary incontinence is an evidence-based treatment option and that, by using it, they will achieve results. To further enhance treatment success, GPs can address their patients’ expectations prior to the start. They can monitor and stimulate their patients to continue usage throughout the eHealth training program by utilizing insights derived from individual log data obtained during the eHealth intervention.

### Supplementary Information

**Supplementary Material 1.**

## Data Availability

Most data generated or analyzed during this study are included in this published article and its supplementary information files. The dataset used and/or analyzed during the current study are available from the corresponding author on reasonable request.

## Abbreviations

GP: General practitioner
ICIQ-LUTSqol: International Consultation on Incontinence Questionnaire for Lower Urinary Tract Symptoms Quality of Life
ICIQ-UI SF: International Consultation on Incontinence Questionnaire Urinary Incontinence Short Form
MSC-12: Mental Component Summary
MUI: Mixed urinary incontinence
OR: Odds ratio
PFMT: Pelvic floor muscle training
PGI-I: Patient Global Impression of Improvement
PCS-12: Physical Component Summary
RCT: Randomized controlled trial
SF-12: Short Form -12
SUI: stress urinary incontinence
