# Supplementary material for: The relation between usage of an eHealth intervention for stress urinary incontinence and treatment outcomes: an observational study
Source: BMC Prim Care. 2024 Mar 16;25:89. doi: 10.1186/s12875-024-02325-4 (PMC10943843; doi:10.1186/s12875-024-02325-4)
Supplement: Supplementary file 1 — Supplementary Material 1. [file 12875_2024_2325_MOESM1_ESM.pdf]

Table S1. Baseline differences between completers and non-completers of T1.

Outcome variable	T1 completed (n=298)	T1 not completed (n=199)	Completed vs non-completed
**Demographic**			
Age, years, mean (SD)	52.6 (11.8)	47.7 (11.8)	P< 0.001*
Educational level, n (%)			P=.617
• Low	26 (8.7)	20 (10.1)	
• High	272 (91.3)	179 (89.9)	
**Medical history**			
Parity 1 > child(ren), n (%)	253 (84.9)	168 (84.4)	P=.885
Postmenopausal, n (%)			P=.005*
• Yes	160 (53.7)	78 (39.2)	
• No	118 (39.6)	100 (50.3)	
• Unknown	20 (6.7)	21 (1.6)	
General quality of life (SF-12), mean (SD)			
• Physical health (PCS-12)	51.0 (7.4)	50.5 (7.6)	P=.480
• Mental health (MCS-12)	49.3 (8.9)	49.0 (9.1)	P=.638
**Incontinence related**			
Incontinence type, n (%)			P=.767
• SUI	244 (81.9)	165 (82.9)	
• MUI	54 (18.1)	34 (17.1)	
Duration <5 years, n (%)	157 (52.7)	122 (61.3)	P=.058
Severity (ICIQ-UI SF), mean (SD)	9.8 (3.2)	10.2 (3.1)	P=.163
Quality of life (ICIQ LUTS-QoL), mean (SD)	31.8 (6.6)	32.5 (7.0)	P=.204
Previous PFMT via therapist[a], n (%)	90 (30.4)	52 (26.4)	P=.336
Previous contact with healthcare professional[a], n (%)	118 (40.5)	75 (37.9)	P=.553
Frequency of pelvic floor muscle exercises at baseline, n (%)			P=0.001*
• Never	116 (38.9)	108 (54.3)	
• <1/week	135 (45.3)	75 (37.7)	
• >1/week	47 (15.8)	16 (8.0)	
Expected ability to train pelvic floor muscles, mean (SD)	7.7 (1.4)	7.6 (1.5)	P=.521
Expected treatment results[a], n (%)			P=.073
• Slight improvement	53 (17.8)	30 (15.2)	
• Major improvement	209 (70.1)	155 (78.3)	
• Cure	36 (12.1)	13 (6.6)	
**User group**			P=0.001*
• Low	120 (40.3)	166 (83.4)	
• Intermediate	101 (33.9)	24 (12.1)	
• High	77 (25.8)	9 (4.5)	

[a] Missing values for the variables previous PFMT via therapist, previous contact with healthcare professional and expected treatment results (n=4, n=8, n=1, respectively).

Table S2. Baseline differences between completers and non-completers of T2

Outcome variable	T2 completed (n=254)	T2 not completed (n=243)	Completed vs non-completed
**Demographic**			
Age, years, mean (SD)	53.6 (11.2)	46.9 (11.8)	P< 0.001*
Educational level, n (%)			P=.531
• Low	25 (9.8)	20 (8.2)	
• High	229 (90.2)	223 (91.8)	
**Medical history**			
Parity 1 > child(ren), n (%)	218 (85.8)	203 (83.5)	P=.479
Postmenopausal, n (%)			P<0.001*
• Yes	145 (57.1)	89 (36.6)	
• No	91 (35.8)	132 (54.3)	
• Unknown	18 (7.1)	22 (9.1)	
General quality of life (SF-12), mean (SD)			
• Physical health (PCS-12)	51.2 (7.0)	50.4 (7.7)	P=.224
• Mental health (MCS-12)	49.2 (9.1)	49.1 (8.9)	P=.888
**Incontinence related**			
Incontinence type, n (%)			P=.733
• SUI	213 (83.9)	201 (82.7)	
• MUI	41 (16.1)	42 (17.3)	
Duration <5 years, n (%)	138 (54.3)	144 (59.3)	P=.268
Severity (ICIQ-UI SF), mean (SD)	9.7 (3.0)	10.2 (3.3)	P=.091
Quality of life (ICIQ LUTS-QoL), mean (SD)	31.5 (6.3)	32.5 (7.3)	P=.108
Previous PFMT via therapist[a], n (%)	77 (30.4)	64 (36.7)	P=.355
Previous contact with healthcare professional[a], n (%)	94 (37.5)	94 (39.5)	P=.642
Frequency of pelvic floor muscle exercises at baseline, n (%)			P=0.008*
• Never	101 (39.8)	122 (50.2)	
• <1/week	110 (43.3)	100 (41.2)	
• >1/week	43 (16.9)	21 (8.6)	
Expected ability to train pelvic floor muscles, mean (SD)	7.7 (1.4)	7.6 (1.5)	P=.277
Expected treatment results[a], n (%)			P=.257
• Slight improvement	39 (15.4)	43 (17.8)	
• Major improvement	183 (72.0)	179 (74.0)	
• Cure	32 (12.6)	20 (8.3)	
**User group**			P<0.001*
• Low	94 (37.0)	196 (80.7)	
• Intermediate	86 (33.9)	37 (15.2)	
• High	74 (29.1)	10 (4.1)	

[a] Missing values for the variables previous PFMT via therapist, previous contact with healthcare professional and expected treatment results (n=4, n=8, n=1, respectively).

Table S3. Unadjusted and adjusted mixed model analyses

a. Symptom severity – user groups

	Unadjusted estimate (SE), 95% CI	Unadjusted P value	Adjusted estimate (SE), 95% CI	Adjusted P value
Timepoint*User group		0.001*		0.001*
- T0*Low	Ref	Ref	Ref	Ref
- T1*Intermediate	-0.26 (0.35), (-1.33, -0.02)	0.451	-0.27 (0.35), (-0.95, 0.42)	0.443
- T1*High	-1.49 (0.38), (-2.24, -0.75)	<0.001*	-1.47 (0.38), (-2.22, -0.72)	<0.001*
- T2*Intermediate	-0.59 (0.38), (-1.33, 0.14)	0.114	-0.62 (0.38), (-1.36, 0.12)	0.103
- T2*High	-1.12 (0.40), (-1.91, -0.34)	0.005*	-1.11 (0.40), (-1.89, -0.33)	0.006*

b. LUTS-QoL - user group

	Unadjusted estimate (SE), 95% CI	Unadjusted P value	Adjusted estimate (SE), 95% CI	Adjusted P value
Timepoint*User group		0.012*		0.014*
- T0*Low	Ref	Ref	Ref	Ref
- T1*Intermediate	0.04 (0.59), (-1.12, 1.21)	0.942	0.07 (0.60), (-1.11, 1.24)	0.912
- T1*High	-1.70 (0.65), (-2.96, -0.43)	0.009*	-1.63 (0.65), (-2.90, -0.35)	0.012*
- T2*Intermediate	-0.93 (0.64), (-2.20, 0.33)	0.146	-0.98 (0.65), (-2.25, 0.29)	0.130
- T2*High	-1.97 (0.68), (-3.30, -0.64)	0.004*	-1.96 (0.68), (-3.29, -0.62)	0.004*

Table S4. Univariate analyses baseline variables - success T1

Outcome variable	Exp (B)	95% CI	P value
**Demographic**			
Age, years	1.03	1.00-1.05	0.021*
**Incontinence related**			
Incontinence type, MUI=0; SUI=1	1.02	0.52-1.99	0.963
Duration (<5 yr=0; >5 yr=1)	0.49	0.29-0.84	0.010*
Severity (ICIQ-UI SF)	0.99	0.91-1.07	0.715
Previous PFMT via therapist[a], (no=0, yes=1)	0.94	0.54-1.66	0.837
Expected ability to train pelvic floor muscles	1.59	1.29-1.96	<0.001*
Expected treatment results			0.017*
• Slight improvement	Ref		
• Major improvement	1.66	0.76-3.63	0.203
• Cure	3.91	1.48-10.35	0.006*

Table S5. Univariate analyses baseline variables-success T2

Outcome variable	Exp (B)	95% CI	P value
**Demographic**			
Age, years	1.01	0.98-1.04	0.463
**Incontinence related**			
Incontinence type, MUI=0; SUI=1	0.78	0.37-1.64	0.513
Duration (<5 yr=0; >5 yr=1)	0.54	0.30-0.96	0.037*
Severity (ICIQ-UI SF)	0.97	0.88-1.06	0.496
Previous PFMT via therapist[a], (no=0, yes=1)	0.95	0.51-1.77	0.881
Expected ability to train pelvic floor muscles	1.50	1.20-1.87	<0.001*
Expected treatment results			0.017*
• Slight improvement	Ref		
• Major improvement	1.85	0.73-4.69	0.197*
• Cure	3.30	1.07-10.18	0.038*
